# Proceedings of Syracuse Hahnemannian Club

**Published:** 1888-09

**Authors:** Frederick Hooker

**Affiliations:** Secretary


### PROCEEDINGS OF THE SYRACUSE HAHNEMANNIAN CLUB.

### I.

At the regular meeting of the Syracuse Hahnemannian Club, held May 25th, were present Drs. Hawley, Hooker, A. B. Kinne, Leggett, Robinson, Sheldon, Schumacher, and True.

Paragraphs 28 to 30, inclusive, of the *Organon* were read.

Dr. Hawley—I would like to ask how this theory (Hahnemann states it as such) differs from the allopathic theory, that no two diseases can exist in the system at the same time? The aim is to produce a drug disease which kills the natural disease, and is then itself extinguished by the reaction of the vital force, thus having the system in a state of health.

Dr. Sheldon—Did not Hahnemann refer only to acute diseases ?

Dr. Leggett—Does not an acute disease cause a suspension of the symptoms of a chronic disease ?

Paragraphs 31 and 32 were then read.

Dr. Hawley—If these forces did attack the health unconditionally and we should have an epidemic of cholera there would not be many of us left. This predisposing cause must be present.

Paragraph 32 was then read.

Dr. Hawley—If this were not true there would be no ground for practicing homœopathically, for there might now and then a man be found that drugs would not make sick.

Paragraph 33 was then read, and the discussion of it led to the question of prophylaxis.

Dr. Hawley—Had two cases of scarlet fever in a family where there were four other children, but there were only the two cases; also, had a case in another family where no other member of the family took it, although no prophylactic was given. I don't see how the efficacy of a prophylactic, as Bell., can be proven. I have not seen but one case of Belladonna scarlet fever in twenty years.

Dr. Hooker—If the indicated remedy be given in a case of scarlet fever, does not that render the disease non-contagious, so far as that particular case is concerned ?

Dr. Leggett—Dr. Kent holds that a disease becomes non-contagious as soon as the simillimum is given.

Dr. Kinne—If there is any prophylactic it must be found in the scales taken during desquamation and triturated with Sacch. lac.

Dr. Sheldon—In one family one child had scarlet fever, and another, who was given Bell.$^{6x}$, night and morning, did not have it for six weeks, when it developed and ran its course.

Dr. Hawley—I have not given a prophylactic for thirty years, and hold that no remedy is prophylactic against any disease.

Dr. Hooker—If it were not so the theory of "specifics" would be true.

The Club then proceeded to the discussion of Mercurius.

Dr. True—Recently got an aggravation in a case of syphilis. There was pain in the testicles and groins ; corroding ulcer at the meatus ; headache ; aggravation at night, with fearful bone pains. Merc.-sol.$^{30}$ aggravated, being in doubt the 3x was given, which increased the aggravation. Stillingia $\theta$ cured.

Dr. Hawley—Case.—Woman aged sixty had a fever when young; received one large dose of Murcury; was salivated, and lost all her hair and her eyebrows. Have never been able to afford her more than temporary relief. She cannot live in an ordinary temperature nor bear the least air, yet she sweats profusely all the time.

Dr. True—Saw a young man who had been mercurialized during typhoid fever. All his joints were anchylosed and his bowels constipated. Aur. removed the constipation, but the anchylosis remains.

---

At the regular meeting of the Syracuse Hahnemannian Club, held June 8th, were present Drs. Hawley, Brewster, A. B. Kinne, and Hooker.

Dr. J. C. Jackson was admitted to membership.

Paragraphs 34 to 40, inclusive, of the *Organon* were read.

Dr. Hawley—Drugs are the most terrible sick-making agents we know anything about. Miasms cannot compare with them. Give every man, woman, and child in this city a good dose of Ipecac. powder and all would be sick, while if all were exposed to marsh miasm or small-pox all would not be sick. At the Soldiers' Rest I had twenty-four cases of typhoid and three deaths, while the allopaths had four cases and three deaths.

The allopathic death-rate in pneumonia is twenty-five per cent., expectant death-rate six per cent., and homœopathic death-rate four to five per cent.

The Club then proceeded to the discussion of water.

Dr. Hawley—Drugs, crude, should be dissolved in Aqua dist., after which potencies may be made with ordinary water, inasmuch as the impurities of the water are not potenized. Have seen good results from the water treatment, and always allow water in any case of disease where there is a desire for it. All methods of treatment that are repugnant to the patient should be avoided.

Case.—Measles; patient very sick; no eruption had appeared. By doctor's orders he was refused water, but when no one was watching him he got out of bed and crawled four rods to a spring, where he drank freely, and then started to crawl back, but fainted. When found he was as red as a boiled lobster, and made a good recovery.

Case.—Cholera; patient in collapse; no water allowed. Patient asked for water, drank all he wanted and got well.

Case.—Cholera; patient in collapse; cold surface; pulseless; vomiting; could not retain medicine. Patient called for water, which he vomited at first, but was soon able to retain. A cold compress was applied to the abdomen, and the patient made a good recovery.

---

At regular meeting, held June 15th, were present Drs. Seward, Hawley, Schumacher, Hooker, and Jackson.

Paragraphs 41 to 46, inclusive, of the *Organon* were read and discussed.

Dr. Seward (paragragh 41)—Cases of mercurialization are the worst we meet.

Dr. Hawley—I believe that the mercurial cachexia is never cured.

---

At the regular meeting, held June 22d, were present Drs. Candee, Schumacher, Robinson, Hooker, Sheldon, and Jackson.

Dr. Schumacher read a paper on Lachesis, and cited the following cases of cure:

CASE.—A middle-aged lady had a varicose ulcer; severe burning pain; dark red edges, not disposed to heal. Ars. no relief; Lach.$^{30}$ pain gone in a week, and the ulcer healed.

CASE.—A farmer, aged sixty, pressive pain in stomach, relieved by eating. Lach. cured.

Dr. Hooker—Case of scarlatina in a child seven years. Pulse, 160, weak, irregular; temperature, 104°; epistaxis of dark blood; throat much inflamed and swollen; glands on left side much tumefied; pain on swallowing, extending to ear; left tonsil covered with false membrane; tendency to right side; aggravation after sleep; eruption fully developed. Lach.$^{30}$ cured in a few days, and relieved immediately.

CASE.—Tonsillitis. Patient usually had several attacks during the winter, almost invariably had the tonsil lanced. Throat much swollen; worse on left side; glands much swollen. Merc-sol., some relief, but the local symptoms soon became aggravated, and there was a tendency of inflammation to right side; and it seemed impossible to prevent the abscess on the left side from breaking. Lach.$^{30}$ caused resolution, and he had no more attacks during the winter.

---

At the regular meeting, held June 29th, were present Drs. True, Robinson, Schumacher, and Hooker.

Dr. True—The first dose of Verat-alb. I ever gave was to a woman with colic after eating ice-cream. She had violent colic, cold extremities, and cold sweat. Verat-alb., one dose, cured. I consider cold sweat, especially on the face, as characteristic of Verat-alb. The color of the stools is not so important, if they are profuse, watery, attended with prostration, cold extremities, and cold sweat. I do not believe it indicated without the cold sweat.

---

At the regular meeting, held July 6th, were present Drs. Brewster, Hawley, Sheldon, True, Hooker, Robinson, Schumacher, Leggett, and Dr. Geo. R. Barnes, of Brooklyn.

Dr. Robinson was appointed Chairman *pro tem.*, and called the meeting to order at 8.45.

On motion of Dr. True, the Club voted to so amend the Constitution as to admit of the election of a Vice-President.

Dr. Robinson was nominated for Vice-President, but withdrew in favor of Dr. True, who was then unanimously elected.

Paragraphs 47 and 48 of the *Organon* were read.

Dr. True—I wish Dr. Hawley would make it clear how drugs act.

Dr. Hawley—To understand Homœopathy we must learn to appreciate the fact that the *invisible is the real*—call it life or what you will. We call it vital force, and this force is liable to be turned out of its normal course by inimical forces, as invisible and intangible as itself. This latter force can only be detached from the vital force by some other force *similar* to it. Hence, when we find a man sick, we seek to find that similar force.

We study our drugs by experiments on the healthy, because in the sick the symptoms of drugs and disease are inextricably mixed.

The allopaths cannot see the aggravation from their large doses, because they do not know the pure symptoms of drugs. *Homœopaths* do not treat the name of a disease but the totality of the symptoms.

Old Dr. Biegler, of Albany, once met in consultation three or four of the best allopaths in the city. When asked to name the disease, he replied: "Stannum!" In relaxed conditions of the bowels the allopaths give a cathartic to "alter the secretions," and following it with Opium, think that the Opium cured, whereas it was the cathartic.

Dr. True—I will ask Dr. Hawley if he considers a teaspoonful of Castor oil homœopathic to diarrhœa?

Dr. Hawley—I consider that it may be homœopathic, but Dr. Hawley wouldn't give it.

Dr. True—I will ask Dr. Leggett to explain the term "sick sensitive?"

Dr. Leggett—Where there is a deep-seated miasm the patient is more susceptible to drugs and to outside influences than he would be were it not for the miasm, and this sensitiveness is in proportion to the depth of the miasm.

Dr. Hawley—I question whether a person in perfect health would be affected by potencies at all.

Adjourned to July 13th.

FREDERICK HOOKER, *Secretary.*

