# Erratum

**Published:** 1888-10

**Authors:** 


### ERRATUM.

The Secretary, in his report of the Syracuse Hahnemannian Club, page 492, line twenty-five, uses the word *non-contagious* instead of *non-infectious*. A misuse of words in such connection gives rise to erroneous ideas.

S. L. G. L.

